# Visco-hyperelastic constitutive modeling of soft tissues based on short and long-term internal variables

**DOI:** 10.1186/s12938-015-0023-7

**Published:** 2015-03-30

**Authors:** Sahand Ahsanizadeh, LePing Li

**Affiliations:** Department of Mechanical and Manufacturing Engineering, University of Calgary, 2500 University Drive NW, Calgary, Alberta T2N 1N4 Canada

**Keywords:** Articular cartilage, Constitutive modeling, Ligament, Strain-rate sensitivity, Viscoelasticity

## Abstract

**Background:**

Differential-type and integral-type formulations are two common approaches in modeling viscoelastic materials. A differential-type theory is often derived from a Helmholtz free energy function and is usually more suitable for the prediction of strain-rate dependent mechanical behavior during rapid loading, while an integral-type theory usually captures stress relaxation more efficiently than a differential-type theory. A modeling approach is needed to predict the viscoelastic responses during both rapid loading and relaxation phases.

**Methods:**

A constitutive modeling methodology based on the short and long-term internal variables was proposed in the present study in order to fully use the better features of the two types of theories. The short-term variables described the loading rate, while the long-term variables involving time constants characterized loading history and stress relaxation.

**Results:**

The application of the methodology was demonstrated with particular formulations for ligament and articular cartilage. Model parameters were calibrated for both tissues with experimental data from the literature. It was found that the proposed model could well predict a wide range of strain-rate dependent load responses during both loading and relaxation phases.

**Conclusion:**

Introducing different internal variables in terms of their time scales reduced the difficulties in the material characterization process and enabled the model to predict the experimental data more accurately, in particular at high strain-rates.

## Background

Biological tissues, such as ligaments and articular cartilages, are viscoelastic, i.e. their mechanical behavior is dependent on the history of deformation. Furthermore, the load response of the tissues can be strain-rate dependent, i.e. a greater stress is produced if a strain is applied at a higher speed. Classical theories of viscoelasticity have been mostly formulated using hereditary integrals in describing the stress or strain response, referred to as the integral-type theories of viscoelasticity. One typical example is the quasi-linear (QLV) theory of viscoelasticity [1] that has been widely adopted for soft biological tissues [2-6]. Examples can also be found in polymer mechanics, such as the modified superposition method, Schapery’s nonlinear theory and Bernstein-Kearsley-Zapas theory [7]. Other integral-type theories of viscoelasticity can be found in a review paper of linear and nonlinear viscoelasticity [8].

Another approach is originated from hyperelasticity in which the stress is obtained from a Helmholtz free energy function [9] that is characterized by a measure of deformation. The history of deformation must be introduced into the energy function to account for viscoelasticity [10]. The viscoelastic response may be decomposed into an elastic response and a viscous response. The elastic response is determined by the external loadings or external variables. The viscous response, however, is determined by internal variables associated with the viscous mechanism in the material, which can be mathematically described by an evolution equation, normally a differential equation. Upon solving the evolution equation, the stress can be obtained in the form of hereditary integrals that share a mathematical analogy with the integral-type theories of viscoelasticity. In the case of a linear evolution equation with fixed time constants, a quasi-linear viscoelastic formula can be obtained. This method has been used in modeling plasticity [11], viscoelasticity [12], damage and growth [13]. An advantage of using internal variables is to establish a physical interpretation and thermodynamically acceptable ground for viscoelasticity.

The integral-type theories of viscoelasticity usually capture the stress relaxation (long-term) more efficiently than the stress response during loading (short-term). The QLV theory, for instance, has been developed for the case of step loading which is not practically possible in experiments. Therefore, modified methods have been introduced for improved mechanical characterization [14-16]. One approach is to characterize the viscoelastic response with different material parameters for the loading and relaxation phases, or short and long-term responses [17]. Some differential-type viscoelastic models, on the other hand, contain decoupled viscous and elastic terms in the Helmholtz free energy function, in which the viscous term characterizes the strain-rate dependent load response and the elastic term describes the equilibrium load response [18,19]. This type of constitutive models provides a good fit to the experimental data during loading phase especially at high strain-rates, but fails to predict the stress relaxation when the strain-rate is nearly zero.

The objective of the present study was to develop an anisotropic viscoelastic model that is capable of predicting both short and long-term responses of strain-rate sensitive viscoelastic materials. Our approach followed a study on human patellar tendons, where the viscous stress in an isotropic model was decomposed into two terms according to their time scales, short or long term [20]. We further developed a general formulation for anisotropic materials and particular formulations for ligament and articular cartilage. Moreover, we introduced a framework of internal variables to describe the short and long-term viscous responses. The short-term internal variable was chosen to be the time-derivative of deformation, whereas the long-term internal variable was obtained from the evolution equation.

## Methods

An anisotropic visco-hyperelastic constitutive model was introduced and characterized for the solid matrix of ligament and articular cartilage. The relevant numerical procedure was developed for the matrix and implemented into the commercial finite element software ABAQUS (Simulia, RI, USA) so that the constitutive model can be used for the stress analysis of general boundary-value problems.

### General formulation of the constitutive model

The deformation gradient is denoted by **F**(**X**) and the right Cauchy-Green deformation tensor by **C** = **F**^*T*^**F**. The Helmholtz free energy function can be written as a function of the current deformation, **C**, the history of deformation, **Ξ**, and the material structure tensor, **N**_0_

1 $$ \Psi =\Psi \left(\mathbf{C},\boldsymbol{\Xi}, {\mathbf{N}}_0\right) $$

The history **Ξ** is referred to as internal variable, because it is associated with the intrinsic material properties. The structure tensor is the tensor product of the unit vector **n**_0_ in the primary material direction, e.g. the fiber direction, **N**_0_ = **n**_0_ ⊗ **n**_0_. This tensor characterizes the anisotropy of the material. We can further introduce short and long-term internal variables to describe the history of deformation. The short-term internal variable describes the rate and history of deformation during the loading phase and can be chosen to be the time derivative of the right Cauchy-Green deformation tensor, **Ċ**. The long-term internal variable, **Γ**, determines the viscoelastic behavior of the material at larger time scales, e.g. during stress relaxation. The Helmholtz free energy function can therefore be written in terms of the short and long-term internal variables:

2 $$ \Psi =\Psi \left(\mathbf{C},\dot{\mathbf{C}},\boldsymbol{\Gamma}, {\mathbf{N}}_0\right) $$

It is further assumed that the elastic and viscous terms can be decoupled following previous studies [10,18,19]:

3 $$ \Psi \left(\mathbf{C},\dot{\mathbf{C}},\boldsymbol{\Gamma}, {\mathbf{N}}_0\right)={\Psi}^e\left(\mathbf{C},{\mathbf{N}}_0\right)+{\Psi}_s^v\left(\mathbf{C},\dot{\mathbf{C}},{\mathbf{N}}_0\right)+{\Psi}_l^v\left(\mathbf{C},\boldsymbol{\Gamma}, {\mathbf{N}}_0\right) $$

The superscripts *e* and *v* stand for elastic and viscoelastic responses respectively; and the subscripts *s* and *l* represent the short and long-term viscous responses respectively.

The viscous response is normally determined by the combined effects of several internal variables. For simplicity, however, the short and long-term internal variables were assumed to be decoupled in the present study: the viscous response during loading phase is solely determined by the short-term internal variable (${\varPsi}_l^v=0$ during loading); and the stress relaxation is only determined by a set of evolutionary mechanisms (${\varPsi}_s^v=0$ during relaxation).

The evolution equation commonly used for viscoelastic materials is in the differential form of viscous stress, **S**^*v*^, and elastic stress, **S**^*e*^ [21-23]:

4 $$ {\dot{\mathbf{S}}}^v+\frac{{\mathbf{S}}^v}{\tau_i}={g}_i{\dot{\mathbf{S}}}^e $$

where *τ*_*i*_ are time constants and *g*_*i*_ are ratios of short-term versus equilibrium stresses (*i* = 1, 2, …). Upon solving this equation, the viscous stress can be obtained as:

5 $$ {\mathbf{S}}^v(t)={g}_i{\displaystyle \underset{0}{\overset{t}{\int }} \exp \left[-\left(t-T\right)/{\tau}_i\right]\ }{\dot{\mathbf{S}}}^e\ dT,\kern1em \left(0\le t\le \delta \right) $$

The time derivative of the elastic stress, **Ṡ**^*e*^, is non-zero during loading phase (0 ≤ *t* ≤ *δ*). Therefore, the viscous response depends on the rate of loading reflected in the elastic stress rate as well as the exponentially reduced relaxation function. Eq (5) is a general form of the viscous stress, so we can use it to determine the short-term viscous stress, ${\mathbf{S}}_s^v(t)\ \left(0\le t\le \delta \right)$. When the loading phase is short, only one time constant *τ*_*i*_ is needed, i.e. one internal variable is used for the short-term response. The long-term viscous stress (*t* > *δ*) may require a few internal variables (time constants) to account for. Each variable contributes a particular weight, *w*_*i*_, that decays at a specific rate associated with a time constant, *τ*_*i*_ . The long-term viscous stress is assumed to depend on the short-term viscous stress at the end of loading phase, ${\mathbf{S}}_{\delta}^v={\mathbf{S}}_s^v\left(t=\delta \right)$ as determined by Eq. (5). Therefore, we introduce the following form of evolution equation:

6 $$ {\dot{\mathbf{S}}}_l^v+\frac{{\mathbf{S}}_l^v}{\tau_i}={w}_i{\mathbf{S}}_{\delta}^v,\kern1em \left(t\ge \delta \right) $$

Here, ${\mathbf{S}}_l^v$ can be considered as the part of the long-term viscous stress contributed by the *i*^th^ internal variable. The long-term viscous response can then be obtained as follows:

7 $$ {\mathbf{S}}_l^v(t)={\displaystyle \sum_i{w}_i}{\mathbf{S}}_{\delta}^v{\displaystyle \underset{\delta }{\overset{t}{\int }} \exp \left[-\left(t-T\right)/{\tau}_i\right]\ }dT,\kern1em \left(t\ge \delta \right), $$

This equation shows the decay of the long-term viscous stress (*t* > *δ*) from the peak stress at the end of the loading phase, ${\mathbf{S}}_{\delta}^v$. It should be noted that *g*_*i*_ in Eq. (4) represents the magnitude of stress relaxation. In the standard linear solid model of viscoelasticity, it can be interpreted as the ratio of the stiffness of the Maxwell body to the stiffness of the elastic body. On the other hand, a *w*_*i*_ reflects the contribution associated with an internal variable to the total response (dimension is 1/time as shown in Eq. (6), *Σw*_*i*_ = 1).

In consistence with Eq. (3), the total stress response of the material can be written as the summation of the elastic stress (**S**^*e*^), short $\left({\mathbf{S}}_s^v\right)$ and long-term $\left({\mathbf{S}}_l^v\right)$ viscous stresses using the same index convention:

8 $$ \mathbf{S}(t)={\mathbf{S}}^e(t)+{\mathbf{S}}_s^v(t)+{\mathbf{S}}_l^v(t) $$

The long-term response is naturally contributed from multiple mechanisms with different time constants *τ*_*i*_ and weights *w*_*i*_. In the examples to follow, the time constants *τ*_*i*_ were considered to be independent of strain. Therefore, a quasi-linear form of viscoelasticity was obtained. However, the time constant can also be defined as a function of strain leading to a fully nonlinear description of viscoelasticity [24].

### Particular formulation for ligaments

The extracellular matrix of ligament is mainly composed of a dense network of collagen fibers mostly aligned in the longitudinal direction of the tissue. This structure forms a transversely isotropic characteristic to the tissue. In the present study, ligament was modeled as a composite material consisting of an isotropic non-fibrillar matrix reinforced by the fiber network. As a ligament is physiologically subjected to tension when the fibers sustain most of the load, only the fibrillar matrix was considered viscoelastic and the non-fibrillar matrix was modeled as elastic. The energy of the non-fibrillar matrix, as indicated by the subscript *m*, is uniquely determined by the deformation, **C**. The energy of the fibrillar matrix, as indicated by the subscript *f*, is also associated with the structure tensor **N**_0_ and the internal variables. Therefore, the Helmholtz free energy for ligaments can be written in the decoupled form as

9 $$ \Psi \left(\mathbf{C},\dot{\mathbf{C}},\boldsymbol{\Gamma}, {\mathbf{N}}_0\right)={\Psi}_m^e\left(\mathbf{C}\right)+{\Psi}_f^e\left(\mathbf{C},{\mathbf{N}}_0\right)+{\Psi}_{s,f}^v\left(\mathbf{C},\dot{\mathbf{C}},{\mathbf{N}}_0\right)+{\Psi}_{l,f}^v\left(\mathbf{C},\Gamma, {\mathbf{N}}_0\right) $$

The non-fibrillar matrix was considered to be Neo-Hookean (first term), while an exponential function was adopted for the hyperelastic part of the fibrillar matrix (second term) [19]. The exponential function describes the stiffening of the tissue at larger strains arisen by the gradual recruitment of collagen fibers during further deformation [25]. Although the collagen fibers show a great stiffness in tension, they cannot sustain compression due to their slenderness. Therefore, a piecewise energy function was employed for the second term in equation (9) that determines whether the collagen fibers contribute to the load support based on their deformation state:

10 $$ \begin{array}{l}{\Psi}^e={\Psi}_m^e+{\Psi}_f^e\\ {}\kern1em =\left\{\begin{array}{l}{a}_1\left({I}_1-3\right),\kern10em \mathrm{if}\kern0.75em {I}_4\le 1\kern0.5em \left(\mathrm{compression}\right)\hfill \\ {}\hfill {a}_1\left({I}_1-3\right)+\frac{a_2}{2{a}_3} \exp \left[{a}_3{\left({I}_4-1\right)}^2\right],\kern0.75em \mathrm{if}\kern0.75em {I}_4>1\kern0.5em \left(\mathrm{tension}\right)\hfill \end{array}\right.\end{array} $$

Here, *I*_1_ is the first invariant of **C** defined as *I*_1_ = **C** : **I** = *C*_*ij*_*I*_*ij*_ = *tr*{**C**}, and *I*_4_ is an invariant defined as *I*_4_ = **C** : **N**_0_. Therefore, *I*_4_ is actually the square of the stretch ratio *λ*_*f*_ in the fiber direction, i.e. ${I}_4={\lambda}_f^2$. As can be seen in this equation, the contribution of the collagen fibers is zero when the tissue is under compression, i.e., ${\varPsi}_f^e=0$ when *I*_4_ ≤ 1. The material constants, *a*_1_, *a*_2_ and *a*_3_, must be positive to ensure the convexity of the function.

The short term viscous function was proposed as follows [19]:

11 $$ {\Psi}_{s,f}^v=\left\{\begin{array}{l}0\\ {}0.5\kern0.5em {a}_4\left({I}_4-1\right) \exp \left[{a}_5{\left({I}_4-1\right)}^2\right]{J}_5, \end{array}\right.\kern1em \begin{array}{c}\hfill \mathrm{if}\kern0.75em {I}_4\le 1\hfill \\ {}\hfill \mathrm{if}\kern0.75em {I}_4>1\hfill \end{array} $$

where *J*_5_ is an invariant defined as *J*_5_ = **Ċ**^2^ : **N**_0_ and *a*_4_ and *a*_5_ are viscous material parameters. As mentioned previously, only collagen fibers were considered viscoelastic and thus, the anisotropic energy functions are written only for the fiber network. Similar to the elastic part, the functions are zero under compression (*I*_4_ ≤ 1). This form of short-term viscous function includes the nonlinearities of the short-term viscous response associated with strain (*I*_4_) by the nonlinear exponential function of *I*_4_.

The second Piola-Kirchhoff stress for elastic and viscous terms are then derived from the energy functions [9,19]:

12 $$ {\mathbf{S}}_m^e=2{a}_1\mathbf{I} $$

13 $$ {\mathbf{S}}_f^e=2{a}_2 \exp \left[{a}_3{\left({I}_4-1\right)}^2\right]\left({I}_4-1\right){\mathbf{N}}_0 $$

14 $$ {\mathbf{S}}_s^v={a}_4\left({I}_4-1\right) \exp \left[{a}_5{\left({I}_4-1\right)}^2\right]\left({\mathbf{N}}_0\dot{\mathbf{C}}+\dot{\mathbf{C}}{\mathbf{N}}_0\right) $$

The total stress can then be written as the summation of the stresses obtained hitherto (Eqs. 12–14) as well as the long-term stress (Eq. 7) as a piecewise function:

15 $$ \mathbf{S}(t)=\left\{\begin{array}{l}\begin{array}{l}p{\mathbf{C}}^{-1}+2{a}_1\mathbf{I}, \kern14.81em \mathrm{if}\kern0.75em {I}_4\le 1\\ {}\ \end{array}\hfill \\ {}\hfill \begin{array}{l}p{\mathbf{C}}^{-1}+2{a}_1\mathbf{I}+2{a}_2 \exp \left[{a}_3{\left({I}_4-1\right)}^2\right]\left({I}_4-1\right){\mathbf{N}}_0+{a}_4\left({I}_4-1\right) \exp \left[{a}_5{\left({I}_4-1\right)}^2\right]\left({\mathbf{N}}_0\dot{\mathbf{C}}+\dot{\mathbf{C}}{\mathbf{N}}_0\right)\\ {}+{\displaystyle \sum_i{w}_i}{\displaystyle {\int}_{\delta}^t \exp \left[-\left(t-T\right)/{\tau}_i\right]{\mathbf{S}}_s^vdT, \kern6.5em \mathrm{if}\kern0.75em {I}_4>1}\end{array}\hfill \end{array}\right. $$

where *p* is a Lagrange multiplier that was introduced to enforce the tissue incompressibility.

The material parameters can be obtained from uniaxial tensile tests. Assuming the *x* direction to be the direction of loading and considering the material to be incompressible, the deformation gradient is of the following form:

16 $$ \mathbf{F}={\left(\lambda,\ \frac{1}{\sqrt{\lambda }},\ \frac{1}{\sqrt{\lambda }}\right)}^T\mathbf{I} $$

from which the right Cauchy-Green deformation tensor can be obtained:

17 $$ \mathbf{C}={\left({\lambda}^2,\ \frac{1}{\lambda },\ \frac{1}{\lambda}\right)}^T\mathbf{I} $$

Using the aforementioned matrix (Eq. 17) along with the structure tensor obtained from the unit vector of fibers ${n}_0=\overrightarrow{i}$ to obtain *I*_4_, the components of the hyperelastic second Piola-Kirchhoff stress will be obtained from Eqs. (12–14):

18 $$ {S}_{11}^e=2{a}_1+p/{\lambda}^2+2{a}_2\left({\lambda}^2-1\right) \exp \left[{a}_3{\left({\lambda}^2-1\right)}^2\right] $$

19 $$ {S}_{22}^e={S}_{33}^e=2{a}_1+p\lambda $$

As there is no stress in the lateral directions, i.e., ${S}_{22}^e={S}_{33}^e=0$, the Lagrange multiplier, *p*, can be calculated as:

20 $$ p=-2{a}_1/\lambda $$

The time derivative of the right Cauchy-Green deformation tensor, **Ċ**, is also needed for calculation of the viscous stresses:

21 $$ \dot{\mathbf{C}}={\left(2\lambda \dot{\lambda},-\dot{\lambda}/{\lambda}^2,-\dot{\lambda}/{\lambda}^2\right)}^T\mathbf{I} $$

The viscous stress can also be obtained from Eq. (14) similarly by substituting the corresponding matrices (Eqs. 17 and 21):

22 $$ {S}_{11}^v=4{a}_4\lambda \left({\lambda}^2-1\right) \exp \left({a}_5{\left({\lambda}^2-1\right)}^2\right)\dot{\lambda} $$

The first Piola-Kirchhoff or nominal stress is commonly used in experimental studies. It can be obtained from the second Piola-Kirchhoff stress according to:

23 $$ \mathbf{P}={J}^{-1}\ \mathbf{F}\ \mathbf{S} $$

Therefore, the nominal stress in the direction of loading was derived for data fit as follows:

24 $$ {P}_{11}=\left\{\begin{array}{l}2{a}_1\left(\lambda -1/{\lambda}^2\right), \kern14em \mathrm{if}\kern0.75em {I}_4\le 1\hfill \\ {}\hfill \begin{array}{l}2{a}_1\left(\lambda -1/{\lambda}^2\right)+2{a}_2\lambda \left({\lambda}^2-1\right) \exp \left[{a}_3{\left({\lambda}^2-1\right)}^2\right]+4{a}_4\left({\lambda}^2-1\right){\lambda}^2 \exp \left[{a}_5{\left({\lambda}^2-1\right)}^2\right]\dot{\lambda}\\ {}+{\displaystyle \sum_{i=1}^N{w}_i{\displaystyle {\int}_{\delta}^t \exp \left[-\left(t-T\right)/{\tau}_i\right]{P}_{11}^vdT, \kern5.5em \mathrm{if}\kern0.75em {I}_4>1}}\end{array}\hfill \end{array}\right. $$

### Particular formulation for articular cartilage

Articular cartilage can be modeled as a fluid-saturated non-fibrillar matrix reinforced by a collagen network [26,27] with various fiber alignments depending on the depth of the tissue and site where the tissue is located in the joint. A thin and narrow strip of cartilage aligned in the fiber direction, as used in uniaxial tensile testing, is similar to a ligament as the fluid pressurization does not affect the tensile properties significantly [28]. The hyperelastic Helmholtz free energy function for cartilage is also composed of isotropic and anisotropic parts representing the non-fibrillar and fibrillar matrices respectively. Similar to the ligament model (Eq. 10), a piece wise function is used to describe the tension-compression difference of the fibers in articular cartilage:

25 $$ {\Psi}^e=\left\{\begin{array}{l}{b}_1\left({I}_1-3\right), \\ {}{b}_1\left({I}_1-3\right)+\frac{1}{2}{b}_2{\left({I}_4-1\right)}^2+\frac{1}{3}{b}_3{\left({I}_4-1\right)}^3, \kern0.5em \end{array}\right.\begin{array}{c}\hfill \mathrm{if}\kern0.75em {I}_4\le 1\hfill \\ {}\hfill \mathrm{if}\kern0.75em {I}_4>1\hfill \end{array} $$

The short-term viscous potential was introduced to resemble Equation (11) as follows:

26 $$ {\Psi}_s^v=\left\{\begin{array}{c}\hfill 0,\kern6.25em \mathrm{if}\kern0.5em {I}_4\le 0\hfill \\ {}\hfill {b}_4\left({I}_4-1\right){J}_5 \ln {I}_4,\kern0.5em \mathrm{if}\kern0.5em {I}_4>0\hfill \end{array}\right. $$

By enforcing incompressibility for the tissue in tensile testing, the second Piola-Kirchhoff stress is obtained:

27 $$ \mathbf{S}(t)=\left\{\begin{array}{l}p{\mathbf{C}}^{-1}+2{b}_1\mathbf{I}, \kern12.4em \mathrm{if}\kern0.75em {I}_4\le 1\hfill \\ {}\hfill \begin{array}{l}p{\mathbf{C}}^{-1}+2{b}_1\mathbf{I}+2{b}_2\left({I}_4-1\right){\mathbf{N}}_0+2{b}_3{\left({I}_4-1\right)}^2{\mathbf{N}}_0+2{b}_4 \ln {I}_4\left({I}_4-1\right)\left({\mathbf{N}}_0\dot{\mathbf{C}}+\dot{\mathbf{C}}{\mathbf{N}}_0\right)\\ {}+{\displaystyle \sum_i{w}_i}{\displaystyle {\int}_{\delta}^t \exp \left[-\left(t-T\right)/{\tau}_i\right]{\mathbf{S}}_s^vdT, \kern4em \mathrm{if}\kern0.75em {I}_4>1}\end{array}\hfill \end{array}\right. $$

This equation is similar to Eq. (15), and *p* is the Lagrange multiplier.

Similar to Eq. (24), the nominal stress in a uniaxial tensile test can be obtained:

28 $$ {P}_{11}=\left\{\begin{array}{l}2{b}_1\left(\lambda -1/{\lambda}^2\right), \kern11.4em \mathrm{if}\kern0.75em {I}_4\le 1\hfill \\ {}\hfill \begin{array}{l}2{b}_1\left(\lambda -1/{\lambda}^2\right)+2{b}_2\lambda \left({\lambda}^2-1\right)+2{b}_3\lambda {\left({\lambda}^2-1\right)}^2+8{b}_4{\lambda}^2 \ln {\lambda}^2\left({\lambda}^2-1\right){\lambda}^2\\ {}+{\displaystyle \sum_i{w}_i}{\displaystyle {\int}_{\delta}^t \exp \left[-\left(t-T\right)/{\tau}_i\right]{P}_{11}^vdT, \kern2.75em \mathrm{if}\kern0.75em {I}_4>1}\end{array}\hfill \end{array}\right. $$

This 1D formulation can then be extended to a 3D formulation of the solid matrix for articular cartilage. Darcy’s law must be incorporated to account for fluid pressure in the tissue under compressive loadings.

### Numerical implementation

The constitutive model proposed here was implemented in the finite element software ABAQUS (Simulia, Providence, RI) by means of a UMAT, a user-defined subroutine in FORTRAN. The constitutive behavior used in ABAQUS for the fluid flow is governed by Forchheimer’s law that includes Darcy’s law as a linear case when the fluid velocities are low. The Newton–Raphson method is used in ABAQUS which requires the stress and the stiffness matrices to be updated in each time step. The Jaumann rate of stress is used in calculations and correspondingly, the Jaumann elasticity tensor along with the Cauchy stress are needed. The Cauchy stress tensor can be obtained by pushing the second Piola-Kirchhoff stress forward [29]:

29 $$ \boldsymbol{\upsigma} ={J}^{-1}\mathbf{F}\ \mathbf{S}\ {\mathbf{F}}^T $$

The elasticity and viscosity tensors can also be derived from the free energy functions or stresses:

30 $$ {\operatorname{C}}^e=2\frac{\partial {\mathbf{S}}^e}{\partial \mathbf{C}}=4\frac{\partial^2{\Psi}^e}{\partial {\mathbf{C}}^2} $$

31 $$ {\operatorname{C}}^v=2\frac{\partial {\mathbf{S}}^v}{\partial \dot{\mathbf{C}}}=4\frac{\partial^2{\Psi}^v}{\partial {\dot{\mathbf{C}}}^2} $$

The elasticity and viscosity tensors were obtained and used to arrive at the Jaumann elasticity tensor following the procedure in [30]. The Jacobian matrix was derived analytically, resulting in a quadratic convergence rate instead of a slower convergence rate associated with the numerical approximation of the Jacobian matrix [31,32].

Reinforcing the incompressibility condition strictly in a numerical procedure can cause problems such as mesh locking. Therefore, a slight compressibility should be introduced into the model which requires the deviatoric-volumetric decomposition of the Helmholtz free energy [33]. For nearly incompressible materials, the decomposition can be applied on both isotropic and anisotropic components of the energy function.

32 $$ \Psi \left({I}_1,{I}_3,{I}_4,{J}_5\right)={\Psi}_{vol}(J)+{\Psi}_m\left({\overline{I}}_1\right)+{\Psi}_f\left({\overline{I}}_4,{\overline{J}}_5\right) $$

Here, *Ψ*_*m*_(*Ī*_1_) is the deviatoric term for the isotropic matrix, and ${\varPsi}_f\left({\overline{I}}_4,{\overline{J}}_5\right)$ for the anisotropic collagen network. The invariants with bar are the invariants of the deviatoric part of the right Cauchy-Green deformation tensor and its time derivative, which are associated with viscoelastic behavior [34].

For the purpose of comparison, the QLV solution below was also numerically implemented following a previously established procedure [27,35]

33 $$ \mathbf{S}(t)={\mathbf{S}}^e(t)+{\displaystyle \sum_i{g}_i}{\displaystyle {\int}_0^t \exp \left[-\left(t-T\right)/{\tau}_i\right]{\dot{\mathbf{S}}}^edT} $$

The data fitting was performed with the least square method using the optimization tool box in MATLAB (MathWorks, MA). Constraints on the parameters were applied whenever applicable. For example, the material properties must be positive to ensure the convexity of the energy functions and positive-definiteness of the elasticity tensors.

## Results

### Data fit for ligaments

**Figure 1:**
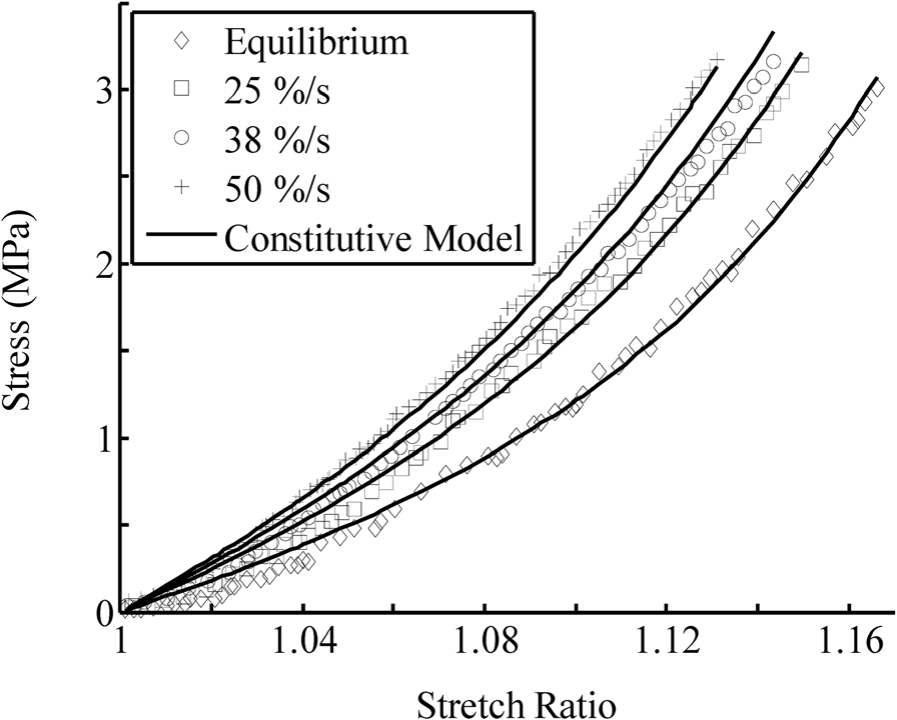
**The model fit for the viscoelastic responses of anterior cruciate ligament under different strain-rates (equilibrium = 1.2%/s).** The material properties used for the numerical simulations (solid curves) were summarized in Table 1. The experimental data were reproduced from the literature [18].

**Figure 2:**
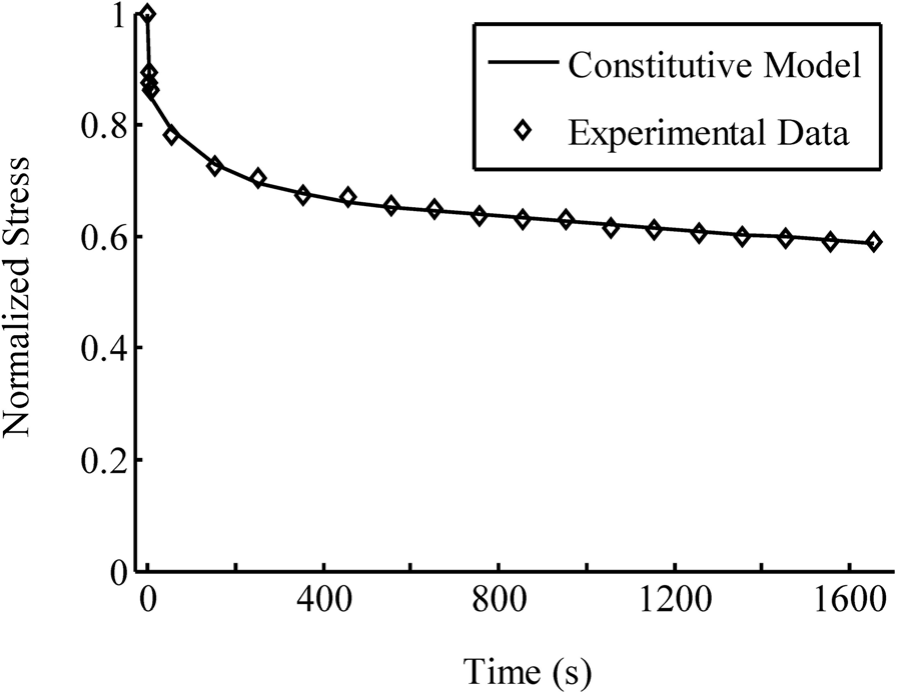
**The model fit for the stress relaxation of anterior cruciate ligament at 16% stretch.** The experimental data were reproduced from the literature [36]. The stress was normalized to the peak stress obtained at the end of ramp loading. A rapid but continuous stress reduction is seen during early relaxation. These data were used to characterize the relaxation parameters, *τ*_*i*_ and *w*_*i*_ , in Table 1.

The tensile testing results of the anterior cruciate ligament were used to calibrate the parameters of the constitutive model. These tests were done under near equilibrium (1.2%/s) and three higher strain-rates of 25%/s, 38%/s and 50%/s [36]. The elastic parameters in Eqs. (12) and (13), *a*_1_, *a*_2_ and *a*_3_, were found by fitting the model to the equilibrium data. The parameter *a*_1_ characterizes the isotropic part of the tissue, and can be obtained from compression tests (which is, however, negligible). The data during ramp loading were used to determine the short-term parameters. The tensile data under 25%/s loading rate were used to find the short-term viscous parameters, *a*_4_ and *a*_5_, which actually predicted the data obtained at the rates of 38 and 50%/s (Figure 1). The stress relaxation response was used for characterizing the long-term viscous parameters, *τ*_1_, *τ*_2_, *τ*_3_, *w*_1_, *w*_2_ and *w*_3_ (Figure 2). The stress was normalized to the peak stress showing the decay of the stress with time after ramp loading. The evaluated parameters of the constitutive equations are summarized in Table 1.

**Table 1:** **The material properties of the anterior cruciate ligament found by fitting the constitutive model (Eq.** 24 **) to tensile experimental data**

**Elastic properties**	**Short-term viscous properties**	**Long-term viscous properties**
*a*_2_ = 2.213 × 10^6^ Pa *a*_3_ = 3.879 (dimensionless)	*a*_4_ = 0.3653 × 10^6^ Pa.s, *a*_5_ = 0.652 (dimensionless)	*τ*_*i*_ = 3.77, 148.24, 10987.98 s
		*w*_*i*_ = 0.154, 0.161, 0.682 s^−1^
		(*i* = 1, 2, 3)

The elastic properties, *a*_2_ and *a*_3_ , were found from the near equilibrium loading (1.2%/s); and the short-term viscous properties, *a*_4_ and *a*_5_ , were determined using the ramp loading under 25%/s strain-rate (Figure 1). Finally, the long term viscous parameters, *τ*_*i*_ and *w*_*i*_, were determined from the stress relaxation data (Figure 2). The compressive property of the ligament, *a*_1_, was neglected in the fit. All parameters were enforced to be positive to satisfy the second law of thermodynamics. Additionally, the constraint of *Σw*_*i*_ = 1 was also applied (Eq. 7) during the optimization.

### Data fit for articular cartilage

**Figure 3:**
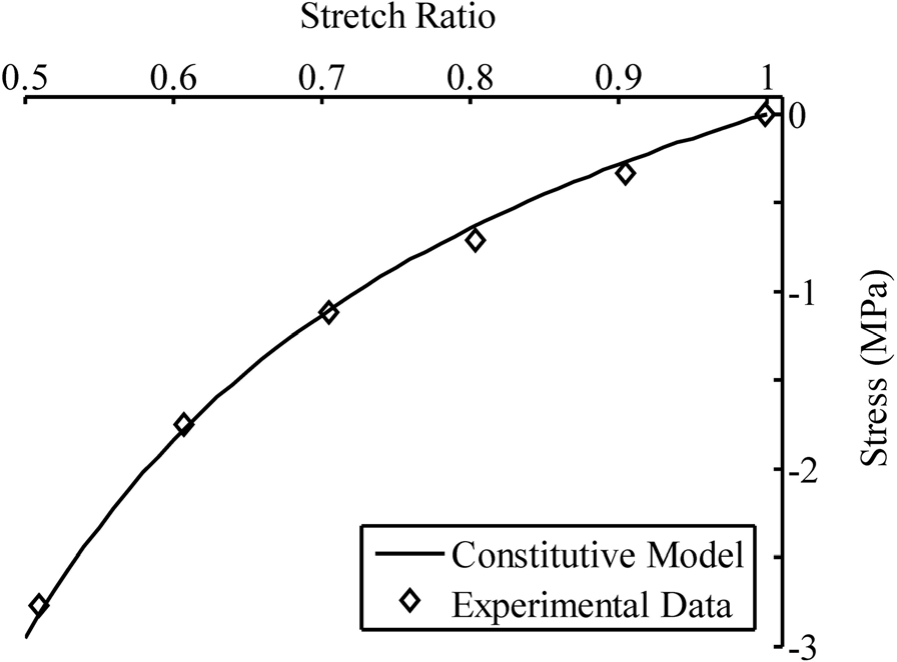
**The model fit for the experimental data of articular cartilage in confined compression at equilibrium [** 38 **].** These data were used to characterize the parameters for the non-fibrillar matrix, *b*_1_ in Table 2.

**Figure 4:**
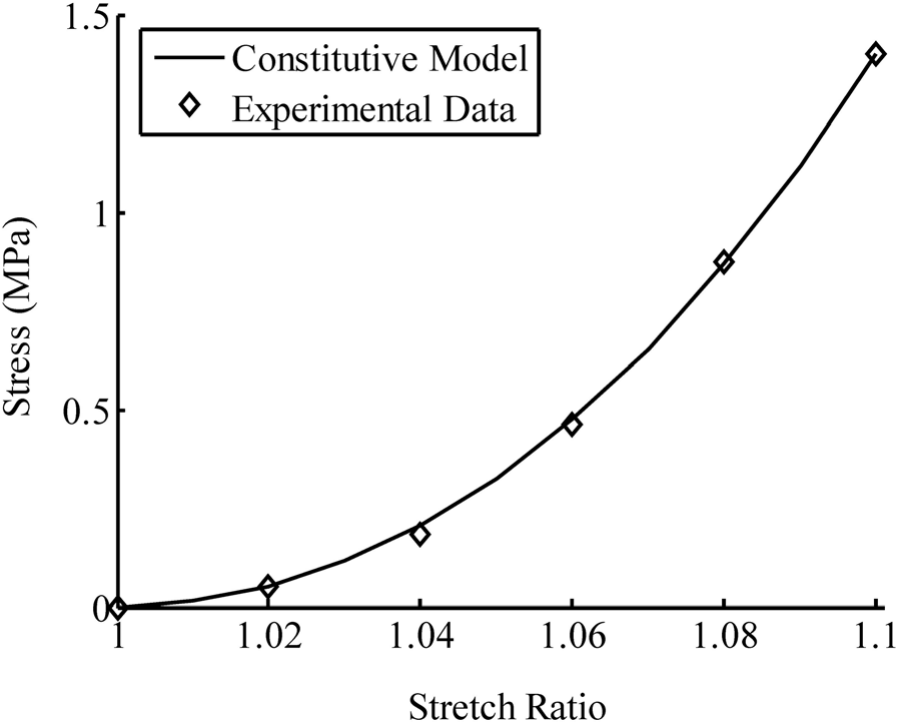
**The model fit for the equilibrium stress in articular cartilage determined from the uniaxial tensile experiments [** 37 **].** These data were used to characterize the elastic parameters, *b*_2_ and *b*_3_, in Table 2.

**Figure 5:**
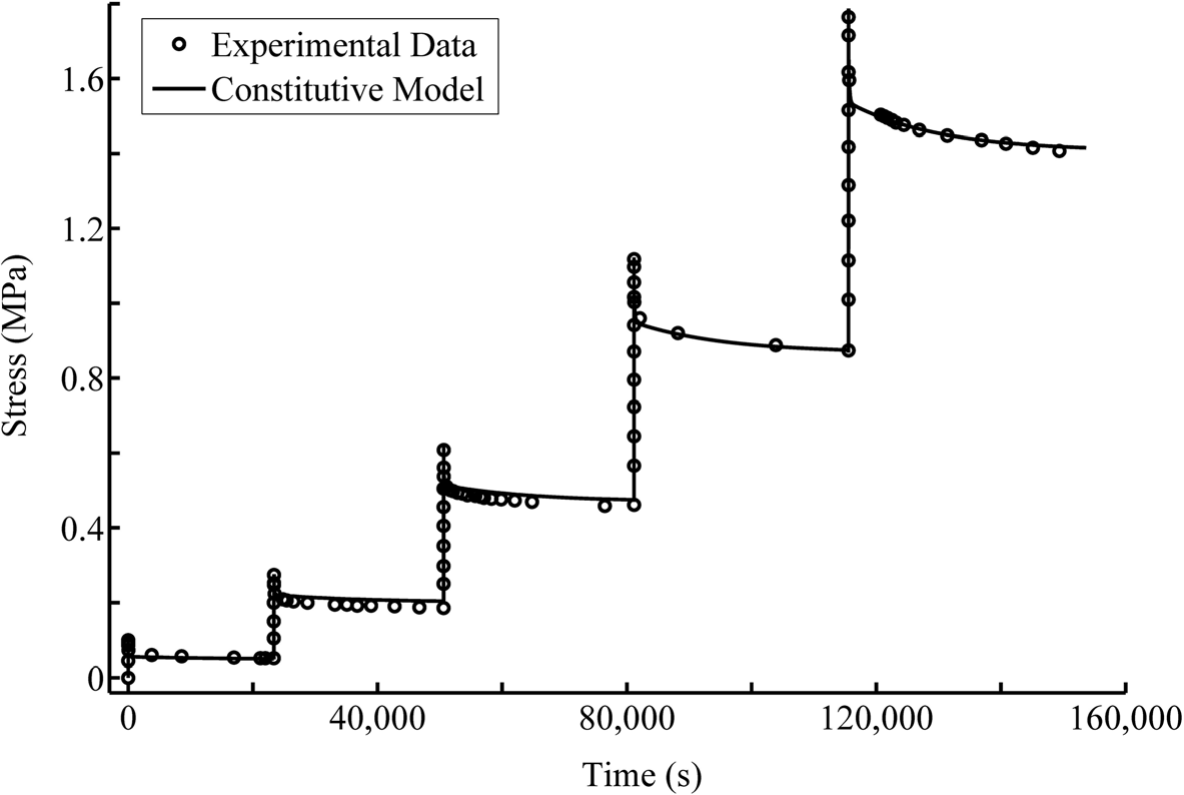
**The model fit for the experimental data of multi-step ramp loading and relaxation of cartilage in tension [** 37 **].** The foregoing test consisted of 5 steps of 2% ramp tension at 0.15%/s followed by stress relaxation. The early relaxation seems to overlap the loading phase because the time involved was very short compared to the large time scale used for the plot. These data were used to characterize the viscous parameters, *b*_4_, *τ*_*i*_ and *w*_*i*_ , in Table 2.

The constitutive model was fit to the 5-step ramp loading and relaxation data from a uniaxial tensile experiment [37] to determine the model parameters (Eqs. 25 and 26). In each ramp loading, a 2% tensile strain was applied at 0.15%/s loading-rate. The ramp loading was followed by a relaxation period that was long enough for the tissue to reach equilibrium completely. In addition, the confined compression test of cartilage [38] was used to evaluate the stiffness of the isotropic non-fibrillar matrix of cartilage, *b*_1_ (Figure 3). The fibers do not play a major role in the confined compression test due to the lateral confinement of the tissue. The stiffness of the collagen fibers, represented by *b*_2_ and *b*_3_, was determined using the equilibrium result of uniaxial tension (Figure 4). Finally, the time-dependent response was used to evaluate the viscous parameters. The ramp loading and stress relaxation phases were used, respectively, to evaluate the short-term (*b*_4_) and long-term viscous parameters (*τ*_1_, *τ*_2_, *τ*_3_, *w*_1_, *w*_2_, *w*_3_). All the material parameters are summarized in Table 2. The fit of the entire test is shown in Figure 5.

**Table 2:** **The material properties of articular cartilage (Eq.** 28 **) found using data obtained from both confined compression and multistep tension and relaxation tests**

**Elastic properties**	**Short-term viscous properties**	**Long-term viscous properties**
*b*_1_ = 0.425 × 10^5^ Pa	*b*_4_ = 190 × 10^6^ Pa.s	*τ*_*i*_ = 141.00, 3.55, 14303.43 s
*b*_2_ = 0.5 × 10^5^ Pa		*w*_*i*_ = 0.346, 0.0709, 0.582 s^−1^
*b*_3_ = 14.2 × 10^6^ Pa		(*i* = 1, 2, 3)

The confined compressive test was used to evaluate the stiffness of the non-fibrillar matrix, *b*_1_ (Figure 3). The tensile test consisted of 5 steps, and 2% tensile strain was applied at 0.15%/s in each step, followed by stress relaxation. The equilibrium data at each step were used to find the elastic stiffness of the fibers, *b*_2_ and *b*_3_ (Figure 4). The short-term, *b*_4_, and the long-term viscous parameters, *τ*_*i*_ and *w*_*i*_, were determined by fitting the model to the entire ramp loading and stress relaxation data (Figure 5). All parameters were enforced to be positive. Additionally, the constraint of *Σw*_*i*_ = 1 was also applied (Eq. 7) during the optimization.

### Strain-rate sensitivity

**Figure 6:**
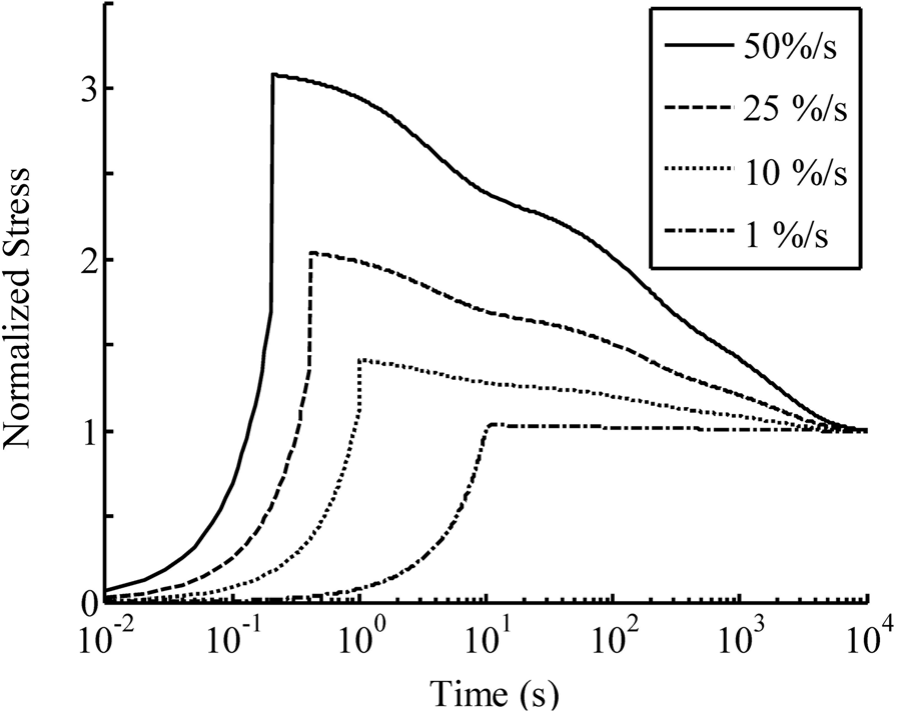
**The model prediction of the ramp loading and relaxation for the ligament under 15% tensile strain at the strain rates of 1, 10, 25 and 50%/s.** The ligament tissue properties were determined from experimental data (Table 1).

**Figure 7:**
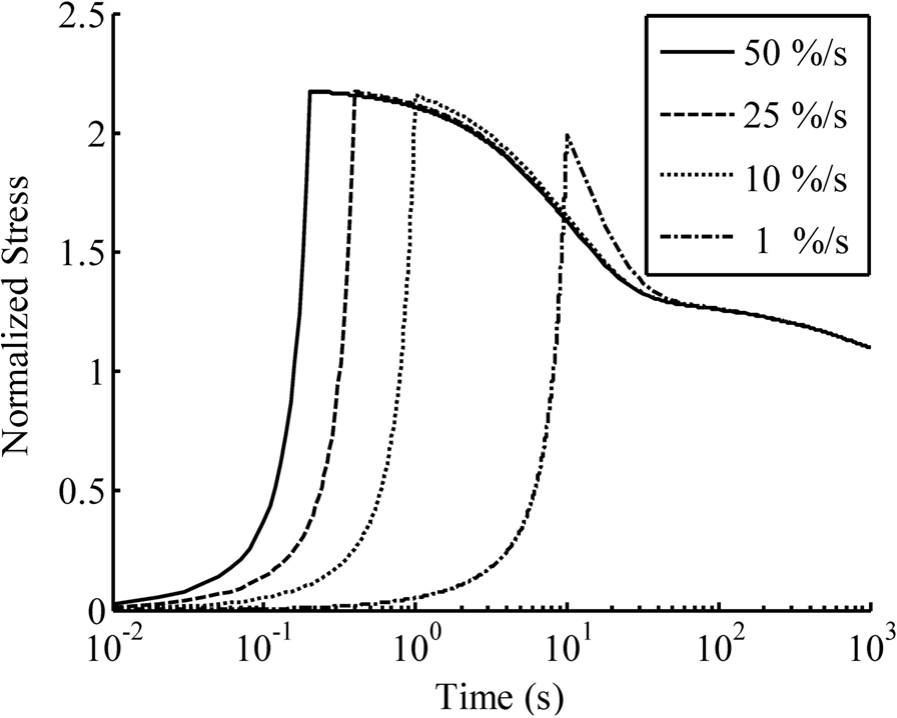
**The model prediction of the ramp tension and relaxation for articular cartilage under 10% tensile strain at various strain rates using a model from the literature (Eq.** **33** **).** The predicted peak stress was almost the same for all strain-rates. The material properties were adopted from the literature [27]: *g*_*i*_ (*i* = 1, 2, or 3) were 0.870, 0.036 and 0.273 (dimensionless) respectively; and *τ*_*i*_ were 10, 100 and 1000 seconds respectively.

A 10% tensile strain was simulated in ligament and cartilage at strain-rates of 1%/s, 10%/s, 25%/s and 50%/s using the material properties obtained from experiments (Tables 1 and 2). The stress response was normalized to the elastic equilibrium stress, so that the ratios of peak stresses relative to the equilibrium stress are clearly demonstrated (Figure 6). The proposed constitutive model produced rate-sensitive results: the peak stress at the highest strain-rate was approximately three times of that at the slowest strain-rate (Figure 6). The peak stress predicted by the QLV theory (Eq. 33) was almost the same for all strain rates (Figure 7).

## Discussion

The model developed in this study was able to predict the viscoelastic behavior in a wide range of strain-rates. For instance, the material properties of ligament were determined from the experimental data obtained at 1.2% and 25%/s strain rates, but were able to capture the data obtained at higher strain rates of 38 and 50% (Figure 1). The strain-rate sensitivity during loading was characterized by the short-term viscous energy function, which can be adequately determined from experimental data in the strain-rate range of interest. The examples presented in the present study were among biological tissues. However, the approach may be equally applicable to other viscoelastic materials such as polymers, hydrogels and in particular to fiber-reinforced anisotropic materials.

The proposed model was also able to account for the stress relaxation response (Figure 2), in addition to the strain-rate dependent behavior during loading discussed above. The differential-type models have been commonly used for soft tissues such as the anterior cruciate ligament [18,39,40], liver [41] and periodontal ligament [42]. They normally fail to predict stress relaxation, and are therefore suggested to be used only for materials with “short-time memory” [41]. This limitation was removed in our modeling by introducing the short and long-term internal variables. The proposed evolution equation (Eq. 6) established the connection and continuity of the short and long-term behaviors which was a key of the approach. The relations between the short and long-term material parameters may also be found using the stress continuity at the end of the loading phase just prior to relaxation (*t* = *δ*) using Eqs. (5) and (7).

The model for articular cartilage was validated against multi-step tension-relaxation data (Figure 5). It is necessary to examine a nonlinear model at different levels of loadings. The multi-step test demonstrated the nonlinearity at both transient and equilibrium responses, i.e. the 5 peak points should present a nonlinear curve, and the 5 end points plus the origin should give another nonlinear curve. A quasi-linear theory may provide a good fit to the relaxation data obtained at one strain, even large, it may fail to account for the relaxation data obtained at another strain. This is because the reduced relaxation function, shown as the exponential integral in Eq. (7), while being a nonlinear function of time, rapidly losses its nonlinearity with time. Therefore, a fully nonlinear formulation including strain dependent time constants may be necessary for some materials.

Fixed time constants, *τ*_*i*_ , were used to obtain example solutions for the evolution equation (Eq. 6), which led to quasi-linearity in stress relaxation (Eq. 7). In other words, the short-term (loading phase) response is fully nonlinear but the long-term (stress relaxation) response is quasi-linear. These simple examples were used to demonstrate the approach of using short and long-term internal variables without adding much complexity to the formulation. However, this limitation can be removed by introducing strain dependent time constants for a better description of the stress relaxation [43] especially at large deformation [44]. Although articular cartilage is often modeled using the quasi-linear QLV theory [45,46], nonlinear theories were shown to be more accurate in fitting the experimental data at different strain-levels [47].

The differential-type viscoelastic modeling approach, often used in modeling ligaments, was extended for articular cartilage in the present study after anisotropic fibril-reinforcement was included in the general framework. The strain-rate sensitivity of the proposed model, however, was not examined for cartilage because of limited availability of tensile data for articular cartilage. The strain-rate dependent load response of cartilage in tension was only investigated in one study using strain-rates of 20, 50 and 70%/s [48]. The tensile modulus was found to increase substantially from the rate of 50%/s to 70%/s. Unfortunately, it was not convenient to use the modulus data to fit the stress in our modeling. Our short-term viscous function for articular cartilage was calibrated using the available experimental data with no variable strain-rates. However, the model should be able to describe the strain-rate dependence of cartilage in tension due to similar tensile load-bearing mechanism in cartilage and ligament. Also for the reason of limited data availability for the strain-rate dependent response, the model capacity in describing hysteresis was not examined in the present study.

The proposed constitutive model can also be applied to strain-rate insensitive viscoelastic materials using the concept of pseudo-elasticity [1]. The pseudo-elastic function used previously predicted the short-term response well, but failed to describe the stress relaxation. A strain-rate insensitive integral-type viscoelastic model was introduced for linear materials only [49]. Within the framework of the present constitutive modeling, the short-term energy function can be replaced with a pseudo-elastic energy function with no or little dependency on strain-rate. This approach would predict the short-term response while at the same time accounting for the stress relaxation.

A major limitation of the present study was limited experimental validation. Only a few simple tensile and compressive tests were used for the curve fit. Loading and unloading scenarios need to be examined to determine the model capacity in describing the hysteresis of viscoelastic materials, as it was successfully done with periodontal ligaments [50]. Biaxial tensile tests may be further used to characterize the model parameters as they may reveal different tensile properties [51], as compared to uniaxial tensile tests. Moreover, the proposed methodology requires separate viscous functions for short-term and long-term responses, which may potentially introduce more model parameters that require various types of test data for the unique determination. A statistical analysis on multiple data fits needs to be performed in order to gain more confidence on the material properties.

## Conclusions

A general constitutive modeling methodology was developed based on the short and long-term internal variables, and examples of anisotropic visco-hyperelastic constitutive models were used to demonstrate the framework. Anisotropic fibril-reinforcement was implemented in order to make it applicable for articular cartilage. The experimental data of ligament and articular cartilage were used to characterize the model parameters. It was found that using both the short and long-term internal variables enhanced the capability of the models to predict both short- and long-term mechanical responses of the tissues especially loaded at high strain-rates. The present study also demonstrated the necessity of fitting multiple model parameters using multiple tissue tests, e.g. using confined compression, simple tensile and multi-step tension-relaxation tests for articular cartilage. The material properties thus determined are more reliable although requiring more test data. Further experimental data are required to validate the material properties presented. The fully validated constitutive models may be used in patient-specific modeling of knee joint to determine the loading rate dependent mechanical function of the joint that is repeatedly subjected to high speed loadings in physiological conditions.
